# Supplementary material for: Diagnostic Errors in Tuberculous Patients: A Multicenter Study from a Developing Country
Source: J Environ Public Health. 2018 Nov 13;2018:1975931. doi: 10.1155/2018/1975931 (PMC6260540; doi:10.1155/2018/1975931)
Supplement: Supplementary Materials — The supplementary table is provided to present several examples of errors in diagnosing the extrapulmonary TB and their consequences. Although diagnosing the complex cases such as those mentioned in this table could be difficult and challenging, hypothesis generation by using analytical diagnostic approach could prevent some of these misdiagnoses. [file 1975931.f1.pdf]

Supplementary. Several examples of TB patients who were experienced diagnostic errors

	Final diagnosis	TB suggestive symptoms	Diagnostic process(es) that was failed	Early consequences
Case 1. A 90-year-old male with new onset of malaise and progressive cognitive impairment had referred to a neurologist. Brain MRI was performed that revealed nothing abnormal. The diagnosis of dementia was made, and patient treated accordingly. Subsequently he developed gait difficulty and urinary incontinence. After visiting another doctor, the diagnosis of UTI was made and patient treated accordingly. Urinalysis showed pyuria, but urine culture was negative. Two other physicians also treated patients with the presumptive diagnosis of UTI in outpatient setting. Chest radiography that performed for evaluation of the reason of tachypnea showed bilateral pulmonary infiltrate. One of the physician in charge interpreted it as old pulmonary fibrosis unrelated to the recent clinical scenario. Three weeks later, he was admitted in the hospital with the impression of nonresponding UTI. Physical examination showed memory impairment, the presence of neurologic deficits including cerebellar ataxia as well as positive meningeal signs. Examination of CSF showed lymphocyte predominant pleocytosis and a few acid-fast bacilli. Moreover, pleural fluid analysis revealed lymphocyte predominant	Disseminated TB, involving CNS, lungs, pleura and urinary system.	Fever and mental confusion of insidious onset	Misinterpreted critical piece of history data; Failure in eliciting a critical physical exam finding; Erroneous clinician interpretation of test; Failure in considering correct diagnosis [Premature closure of diagnosis; anchoring bias; diagnostic momentum]	Delayed diagnosis; starting treatment at more advanced stages of disease

exudate. Acid fast bacilli were also evident on urine smear examination.				
Case 2. An 80-year-old male had referred to a urologist due to testicular swelling and had undergone orchiectomy to verify or rule out the diagnosis of testicular cancer. He had come back to another physician after six months with complaints of fever, coughing, and weight loss. The chest radiography revealed infiltration in the lower zone of the right lung. In accordance with the history taking of the patient, recent symptoms had been appeared since several months before the onset of testicular swelling, but with low severity and more severe then. In addition, the patient did not follow-up the result of the histopathology of the testicular tumor and the health-care system had not informed him about the test result. The result of histopathological examination of testicular biopsy specimen found to be reported as granulomatous inflammation with caseous necrosis. Moreover, his sputum smear examination was positive for acid-fast bacilli.		persistent cough for two weeks or more	Failure in eliciting a critical piece of history data;<br>Delayed follow-up action on test result;<br>Failure scheduling ordered referral	Delayed diagnosis; increased period of infectivity and consequently increased transmission of disease
Case 3. A 19-year-old boy was referred to a gastroenterologist because of diarrhea and was admitted to hospital for diagnostic evaluation with the initial impression of the inflammatory bowel disease.<br>Stool exam revealed fecal leukocytes. CT scan of the abdomen and pelvis showed	Disseminated TB. Involving lungs and GI tract	Loss of appetite and weight, abdominal pain, diarrhea or constipation, mass in the abdomen, fluid in the abdominal cavity	Delay in eliciting a critical piece of history data	Delayed diagnosis; increased period of infectivity and consequently increased transmission of disease; substantial exposure of other patients and

bowel wall thickening in the terminal ileum. He underwent a colonoscopy and biopsy. The histopathological examination of the specimen revealed granulomatous inflammation with acidophilic necrosis. At this<br>time, the patient was questioned on respiratory symptoms and the history of close contact with a TB patient. The answer to second question was positive. In response to the first question, he states, "I had cough and sputum production since one or two months before diarrhea". Therefore, chest radiography was ordered; the results indicated bilateral apical fibro cavitary involvement. The sputum smear examination revealed 3+ positive result. During the two weeks pending the etiologic diagnosis, he was admitted in a 6-bed room of hospital without implementation of any precaution to prevent disease transmission.		(ascites).<br><br>Persistent cough for two weeks or more		healthcare workers to patient's respiratory secretion
Case 4. A 36-year-old man visited an internist with the complaint of subacute progressive ankle pain and swelling, as well as subcutaneous nodules on the dorsum of his left foot and medial of his left ankle. The physician treated the patient with anti-inflammatory drugs, including oral corticosteroid, and methotrexate, based on non-definitive rheumatologic test results including positive RF without aspiration/biopsy of the soft tissue nodules. One month later,	Disseminated TB, involving meninges, lungs, pleura, ankle joints and soft tissue	Localized pain and/or swelling, discharge of pus, muscle weakness, paralysis, stiffness of joints.	Failure/delay in considering correct diagnosis;<br>Failure in ordering referral;<br>Failure in timely follow-up/ rechecking of patient;<br>Failure in considering correct diagnosis<br>[Premature closure of diagnosis; Confirmation bias]	Delayed diagnosis; starting treatment at more advanced stages of disease; multiple organ involvement (disseminated form of TB); CNS involvement

the symptoms continued, and he developed high grade fever, non-productive cough, and subsequently severe headache. Thus, he admitted to the hospital for diagnostic evaluation. On physical examination he was febrile and tachypneic and had meningeal signs. Chest radiography revealed bilateral pulmonary involvement. The content of tissue masses was aspirated that revealed inflammatory cells as well as few acid-fast bacilli. CSF examination revealed lymphocyte predominant pleocytosis, hypoglycorrhachia, and elevated level of protein.				
Case 5. A 65-year-old Afghan man visited a physician with the complaint of chronic respiratory symptoms including cough and progressive dyspnea. The physician ordered a chest radiograph, which showed bilateral micronodular pattern. The diagnosis of sarcoidosis was proposed based on three negative sputum smears and patient was prescribed high doses of oral corticosteroid. Four weeks later, he was admitted to the hospital with respiratory failure and coma. CSF examination showed pleocytosis as well as a few acid-fast bacilli. In addition, smear of stomach fluid also revealed acid fast bacilli. Unfortunately, he died three days after hospitalization despite starting anti-TB medications due to TB-associated sepsis and respiratory failure.	Miliary TB	persistent cough for two weeks or more	Too much weight to lower priority diagnosis; Suboptimal test sequencing; Failure to recognize urgency; Failure in considering correct diagnosis [Premature closure of diagnosis; Confirmation bias]	Delayed diagnosis; starting treatment at more advanced stages of disease; multiple organ involvement (disseminated form of TB); TB-associated sepsis and respiratory failure; death

Case 6. A 21-year-old Afghan girl visited a physician with a complaint of chronic non-productive cough and constitutional symptoms and was introduced to the TB/health center regarding the miliary pattern of infiltrate on chest radiograph. The physician at the TB/health center said, "Go and come back to test whenever you had sputum". She did not visit the TB/health center because she did not have any sputum production during the next several weeks. One month after the visit, the patient was hospitalized due to decreased level of consciousness. At that time, she became a candidate for ventriculoperitoneal shunt placement because of severe hydrocephalus	Miliary TB	Persistent cough for two weeks or more	Suboptimal test sequencing; Failure to refer patient to safe setting/monitoring; Failure to recognize urgency	Delayed diagnosis; CNS involvement; progressive hydrocephalus requiring surgical shunting

MRI: magnetic resonance imaging; UTI: urinary tract infection; CSF: cerebrospinal fluid; TB: tuberculosis; CNS: central nervous system; CT scan: computed tomography; GI: gastrointestinal.
